# Mitral valve repair, how to make volume not matter; techniques, tendencies, and outcomes, a single center experience

**DOI:** 10.1186/s13019-018-0789-3

**Published:** 2018-10-16

**Authors:** Manuel Giraldo-Grueso, Néstor Sandoval-Reyes, Jaime Camacho, Ivonne Pineda, Juan P. Umaña

**Affiliations:** 1 Vascular Function Research Laboratory, Fundación Cardioinfantil- Instituto de Cardiologia, Bogotá, Colombia; 2 Cardiac Surgery, Fundación Cardioinfantil- Instituto de Cardiologia, Bogotá, Colombia; 3 Cardiac Surgery Department, Fundación Cardioinfantil- Instituto de Cardiologia, Bogotá, Colombia; 4 Director Cardiovascular Medicine, Cardiac Surgery Department, Fundación Cardioinfantil- Instituto de Cardiologia, Bogotá, Colombia

**Keywords:** Mitral regurgitation, Mitral valve annulus repair, Prolapsed mitral valve

## Abstract

**Background:**

Recent evidence has showed us that quality of mitral valve repair is strongly related to volume. However, this study shows how low-volume centers can achieve results in mitral valve repair surgery comparable to those reported by referral centers. It compares outcomes of mitral valve repair using resection versus noresection techniques, tendencies, and rates of repair.

**Methods:**

Between 2004 and 2017, 200 patients underwent mitral valve repair for degenerative mitral valve disease at Fundación Cardioinfantil-Institute of Cardiology. Fifty-eight (29%) patients underwent resection and 142 (71%) noresection.

**Results:**

Follow-up was 94% complete, mean follow-up time was 2.3 years. There was no 30-day mortality. Five patients required mitral valve replacement after an average of 5.3 years (Resection = 2; Noresection = 3). Freedom from severe mitral regurgitation was 98% at 6.6 years of follow-up for the noresection group, and 92.5% at 7 years for the resection group (log rank: 0.888). At last follow-up, two patients died of cardiovascular disease related to mitral valve, 181 patients (86%) showed no or grade I mitral regurgitation. Patients with previous myocardial infarction had increased risk of recurrent mitral regurgitation (*p* = 0,030). Within four years, we inverted the proportion of mitral valve replacement and repair, and in 2016 we achieved a mitral valve repair rate of 96%.

**Conclusion:**

This study suggests that resection and noresection techniques are safe and effective. Recurrence of severe mitral regurgitation and need for mitral valve replacement are rare. We show that low-volume centers can achieve results comparable to those reported worldwide by establishing a mitral valve repair team. We encourage hospitals to follow this model of mitral valve repair program to decrease the proportion of mitral valve replacement, while increasing mitral valve repair.

## Background

Mitral valve repair (MVr) is the gold standard for the treatment of mitral regurgitation (MR) secondary to degenerative mitral valve (MV) disease. MVr was initially performed by Alain Carpentier in 1983, who developed a standardized approach to correct MR, dubbed “the French correction”. It involved leaflet resection followed by annular plication with or without sliding plasty in order to restore the coaptation surface [1]. Excellent, reproducible results led to this technique becoming the gold standard to treat mitral valve prolapse. In 1998, Tirone David et al. proposed a novel repair technique using extended polytetrafluoroethylene (ePTFE) sutures for chordal replacement, preserving leaflet tissue and improving surface of coaptation [2].

Subsequent studies have shown excellent results for both techniques in terms of mortality, morbidity, and freedom from recurrent MR [3]. Controversy remains as to which technique is superior given lack of long-term follow-up with creation of neochordae and the perception that this technique is more difficutl to standardize, preventing widespread application.

In Latin America, long-term results of MVr remain unknown and the established practice is to replace rather than repair the MV. The present study was carried out to evaluate the short and long-term results of MVr using resection (R) versus noresection (NR) techniques in a low-volume center and resolve if a low-volume center can achieve MVr results comparable to those reported worldwide. We analyzed freedom from reoperation, recurrent MR, and functional status, as well as the change in the tendency of MVr and mitral valve replacement (MVR) at our institution over the study period. The findings of the study seek to improve cardiac surgery.

## Methods

### Patients

From January of 2004 to June 2017, 200 patients underwent MVr due to degenerative MV disease at Fundación Cardioinfantil- Institute of Cardiology, in Bogotá Colombia. Patients were identified through an institutional cardiac surgery database. Operational definitions, demographic variables, preoperative, intraoperative characteristics, and 30-day outcomes were obtained retrospectively according to the Society of Thoracic Surgeons database guidelines [4].

Fifty-eight patients (29%) were in the R group and 142 (71%) in the NR group (chordal replacement or just ring annuloplasty). Twelve patients (6%) were lost to follow up.

### Interventions

**Fig. 1:**
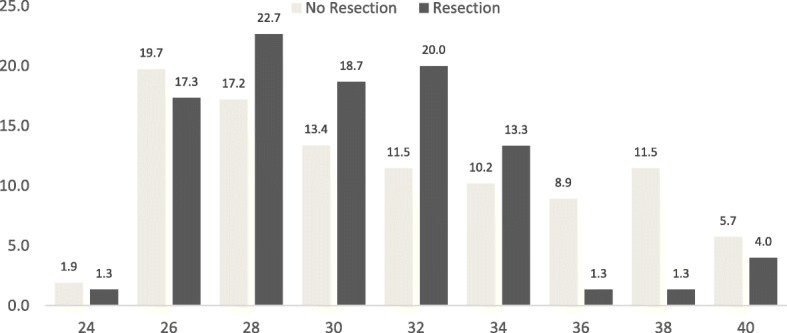
Size of annuloplasty ring. The figure shows the size of annuloplasty ring by resection group. Size of the ring is depicted on de y-axis and the percentage on the x-axis

Operations were performed through a conventional median sternotomy or minimally invasive techniques (right lateral minithoracotomy or periareolar approach). In the conventional approach, cardiopulmonary bypass was established through standard bicaval and aortic cannulation with moderate hypothermia. Intraoperative transesophageal echocardiography was used routinely in all patients. Access to the MV was performed through a left atriotomy. Next, segmental analysis of the MV was performed as described by Carpentier and colleagues [5]. In all patients, ring annuloplasty was performed with a semi-rigid, complete ring Fig. 1.

When the repair was performed minimally invasively, the femoral vessels were cannulated using modified Seldinger technique under echocardiographic guidance. A Chitwood clamp was used and cardiac arrest achieved using HTK or Del Nido cardioplegia. Video assistance was used routinely.

Chordal replacement was performed with 5.0 ePTFE sutures without pledgets, passed as a figure of eight through the tip of the papillary muscle, followed by a figure of eight through the free edge of the prolapsing segment. A minimum of two neochordae were placed, and sutures were added depending on the size of the prolapsing segment. The height of the neochordae was established by filling the ventricle with a cold cardioplegic solution to test the valve hydrostatically. The number of neochordae ranged from one to seven pairs (mean: 1.88). A single pair of neochordae was used in 29% and multiple in 71%. The decision to perform either a R or NR technique was left to the surgeon’s criteria.

Surgical data were obtained by systematic chart review, emphasizing the MVr technique and approach.

### Data collection

Preoperative (age, previous cardiac operation, functional class, Euroscore II, left ventricular ejection fraction, previous arrhythmia, and medical history) and postoperative variables (length of stay, cross-clamp and cardiopulmonary bypass time, reoperation for bleeding and 30-day mortality) were described.

Follow up was performed by telephone or in person (clinic visits). Endpoints were recurrent MR, reoperation or death. Echocardiographic evaluations were performed postoperatively before discharge, 30 to 90 days after surgery, then annually thereafter. The severity of MR was classified as none/trivial (0), mild (I), moderate (II) or severe (III). New York Heart Association (NYHA) functional class was assessed in all the patients. Echocardiographic data were used for analysis only if there were at least two echocardiographic reports available.

We described tendencies and number of cases of MVR and MVr for degenerative MV disease from 2004 to 2016. Data were obtained from the institutional cardiac surgery database.

### Statistical analysis

Baseline demographics and clinical characteristics were summarized using descriptive statistics. For continuous variables, data were presented as mean or median and standard deviations or interquartile range. Categorical variables were presented as absolute numbers and percentages. The frequency of MR was described. The difference between the groups R and NR were ascertained using chi-square test or Fisher test, and Mann-Whitney U test. The endpoint of interest was recurrent severe MR, MV reoperation or death. Patients that did not reach the endpoint were censored at the end of study time. Survival was analyzed through Kaplan-Meier method; the log-rank test was used to determine differences between groups. Statistical analysis was done with Stata SE 14 (program). A significance level of 0.05 was used throughout the analysis.

## Results

### Demographic data

Follow-up was 94% complete with a mean time of 2.33 years. Preoperative variables are summarized in Table 1. Of all patients, 122(61%) were male, and the average age at operation was 58 (48–58) years for the NR group and 56 (50–65) years for the R group. Before surgery, NYHA functional class was assessed in all the patients, 21 (10.5%) were in NYHA class I, 135 (67,5%) class II and 33 (16,5%) class III. Three (1.5%) patients had a history of myocardial infarction before surgery, all of them belong to the NR group. We found differences in the left ventricular ejection fraction (LVEF) between groups; 55% (50–60%) and 60% (51–65) for the NR group and R group, respectively (*p* = 0.013).

**Table 1:** Preoperative, clinical, and perioperative variables of the patients

Variable *n* (%)	No resection *n* = 142	Resection *n* = 58	*P* value
Preoperative variables			
Male sex	83 (58.4)	39 (67,2)	0,247
Age years, median IQR	58 (48–66)	56 (48–66)	0,969
Diabetes	9 (6,3)	1 (1,7)	0,287
Dyslipidemia	18 (12,7)	11 (18,9)	0,252
Dialysis	2 (1,4)	3 (5,2)	0,147
Hypertension	59 (41,5)	20 (34,5)	0,354
COPD	7 (4,9)	4 (6,9)	0,580
Creatinine	1 (0,9-1,08)	0,95 (0,9–1)	0,821
Previous myocardial infarction	0	3 (5,2)	0,023
Previous cardiac operation	4 (2.8)	1 (1,7)	0,999
NYHA functional class			0,079
I	12 (8,7)	9 (17,3)	
II	99 (72,3)	36 (69,2)	
III	26 (19)	7 (13,5)	
Previous arrhythmia	48 (33,8)	19 (32,8)	0,887
LVEF, median IQR	55 (50–60)	60 (51–65)	0,013
Perioperative variables			
Isolated ring annuloplasty	14 (9,8)	0 (0,0)	< 0,001
Isolated MV repair	107 (75)	49 (84,5)	0,108
Non-Isolated MV repair	35 (25)	9 (15,5)	0,235
ASD closure	7 (4,9)	0 (0,0)	0,086
Tricuspid repair	24 (16,9)	9 (15,5)	0,809
Tricuspid replacement	1 (0,7)	0 (0,0)	0,001
Tricuspid repair+ASD closure	3 (2,1)	0 (0,0)	0,013
Minimally invasive	51 (35,9)	7 (12.1)	< 0,001
ICU stay days	1 (1–4)	1 (1–3)	0,495
Post ICU stay (days)	3 (2–5)	4 (3–5)	0,674
Degenerative MV pathology			
Posterior leaflet prolapse	47 (33,1)	35 (60,3)	0,004
Anterior leaflet prolapse	23 (16,1)	4 (6,8)	0,079
Bileaflet prolapse	17 (11,9)	3 (5,1)	0,144
Elongated/ruptured chord(s)	29 (20,4)	10 (17,2)	0,604
Annular dilation	25 (17,6)	2 (3,4)	0,014
Unknown	1 (0,7)	4 (6,9)	0,011
Postoperative complications			
Reoperation for bleeding	0 (0,0)	2 (3,4)	0,083
Renal impairment	2 (1,4)	0 (0,0)	0,503
Hospital length of stay	8 (5–15)	8 (5–14)	0,906
Mortality 30 days	0 (0,0)	0 (0,0)	

Categorical data are expressed as number (%) and continuous data as median (Interquartile range)

*COPD* Chronic Obstructive Pulmonary Disease, *ICU* Intensive Care Unit, *IQR* Interquartile Range, *LVEF* Left Ventricular Ejection Fraction, *NYHA* New York Hear Association

**Fig. 2:**
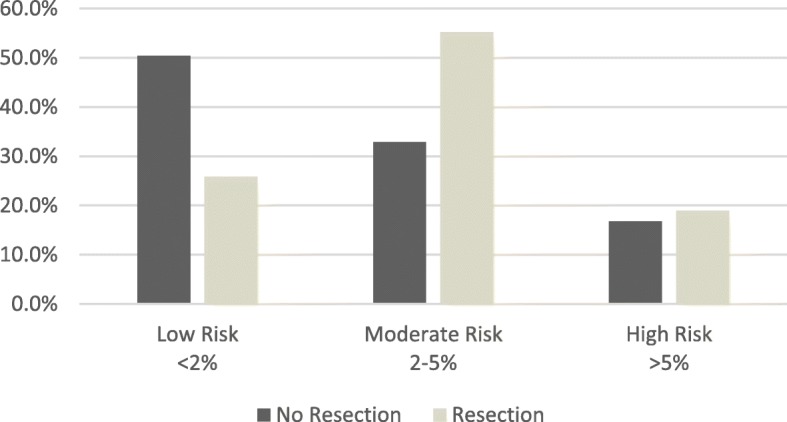
Preoperative Euroscore II risk assessment. Figure shows Euroscore II risk assessment in NR and R groups

Euroscore II was calculated in all patients before surgery. 50.4% in the NR group were classified as low risk, compared to 25,9% in the R group (risk < 2%) Fig. 2.

### Perioperative outcomes

Perioperative variables are summarized in Table 1. One hundred and seven patients (75%) of the NR group and 48 (84.5%) of the R group underwent isolated MVr. Mean cardiopulmonary bypass time was similar for both groups, 117 min (IQR 95–141) and 117 min (IQR 105–143) for the NR and R groups respectively. Forty-seven (33.1%) patients from NR group and 35 (60%) from R group had a posterior leaflet prolapse (*p* = 0,004). There was a statistically significant difference in the number of minimally invasive procedures performed in each group, with 51 (32.9%) in the NR group and 7 (12.1%) in the R group (*p* = 0.001). Overall 30-day mortality was 0%.

### Survival outcomes

NYHA class and incidence of MR at last follow-up in 188 patients are reported in Table 2. Functional class was assessed in all the patients, most of whom showed significant improvement: 156 (83%) had NYHA class I, 25 (13%) class II, 5 (3%) class III and 2 (1%) class IV. Patients in NYHA class IV had concomitant chronic obstructive pulmonary disease (COPD). Ninety-eight patients (52%) had none/trace MR, mild MR in 70 (37%), and moderate/severe in 20 (10%).

**Table 2:** Postoperative occurrence of mitral regurgitation and assessment of NYHA class

Variable	No resection *n* = 136	Resection *n* = 52	*P* value
NYHA functional class			0.797
I	115 (84.5)	41 (78.8)	
II	16 (11.7)	9 (17.3)	
III	3 (2.2)	2 (3.8)	
IV	2 (1.5)	0	
Mitral valve regurgitation			0.267
None/Trace	76 (56.0)	22 (42.3)	
Mild	48 (35.3)	22 (42.3)	
Moderate	9 (6.6)	6 (11.5)	
Severe	3 (2.1)	2 (3.8)	

Categorical data are expressed as number (%)

*NYHA* New York Hear Association

**Fig. 3:**
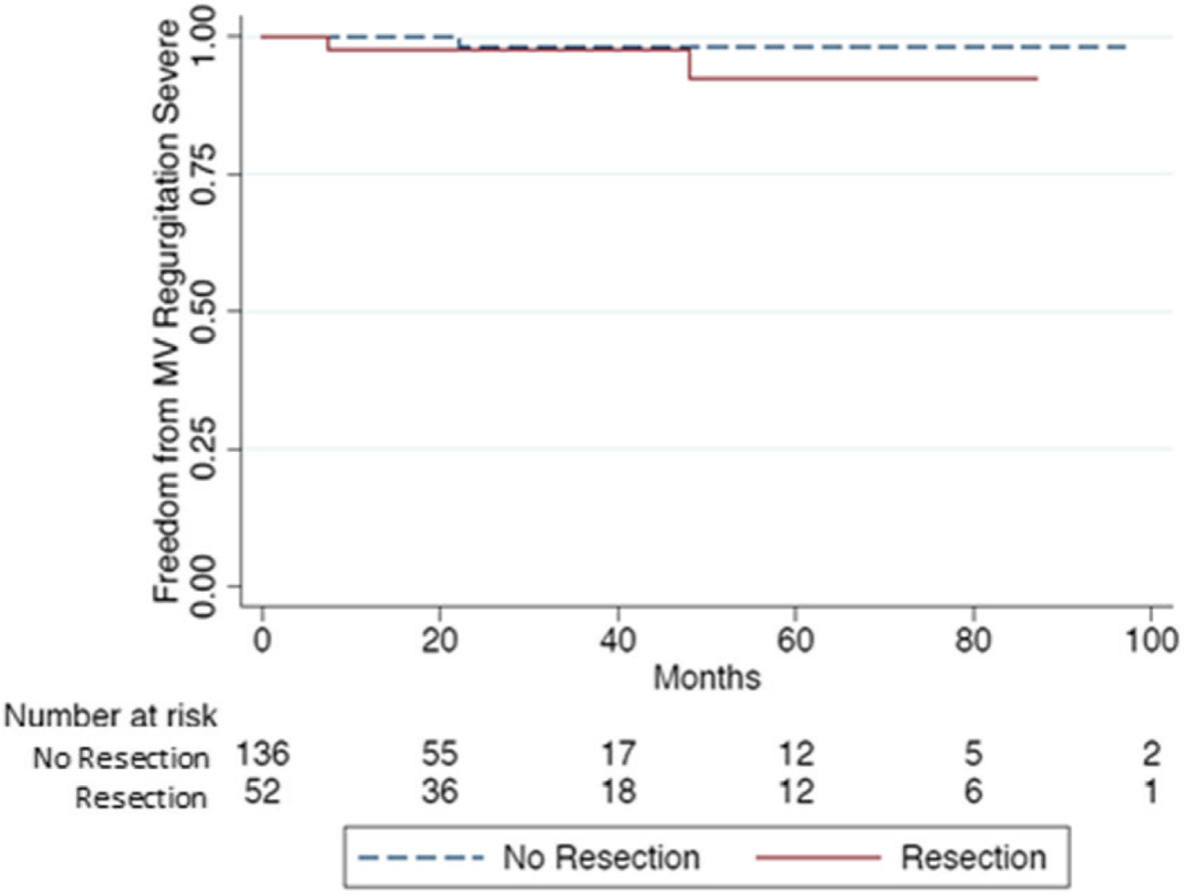
Freedom from > 3 Mitral Regurgitation. Kaplan-Meier estimate of survival function from at least > 3 mitral valve regurgitation for 188 patients with degenerative mitral valve disease

There were only two cardiac-related deaths at last follow-up. Freedom for severe MR was 98% at 6.6 years of follow-up for the NR group, and 92.5% at 7 years of follow-up for the R group. Based on MVr technique, patients in the R group had the same likelihood of developing MR compared to patients in NR group (log rank: 0.881). Five patients required an MV replacement after an average of 5.3 years, 3 belonged to the NR group and 2 to the R group Fig. 3.

### Bivariate analysis

In the bivariate analysis, patients with previous myocardial infarction had an increased risk of developing at least moderate recurrent MR (*p* = 0,030). Preoperative variables such as diabetes, dialysis, dyslipidemia, hypertension and previous arrhythmia, were not associated with an increased risk of developing recurrent MR after MVr. Patients that underwent minimally invasive repair, had a lower risk of developing recurrent MR (*p* = 0,040) Table 3.

**Table 3:** Bivariate analysis identifying factors related to at least moderate MV regurgitation in 188 patients

Bivariate analysis	OR	CI 95%	*P* value
Previous myocardial infarction	18,55	1602-214,857	0,030
Diabetes	0,93	0.112–7.747	1000
Dialysis	2,15	0.03–20,314	0,043
Minimally invasive technique	0,22	0,051-1019	0,040
Dyslipidemia	1,65	0.508–5.420	0,489
Hypertension	1,29	0,509-3299	0,585
Arrhythmia	1,01	0,294-3518	1000

### Mitral valve surgery tendencies and repair rate

**Fig. 4:**
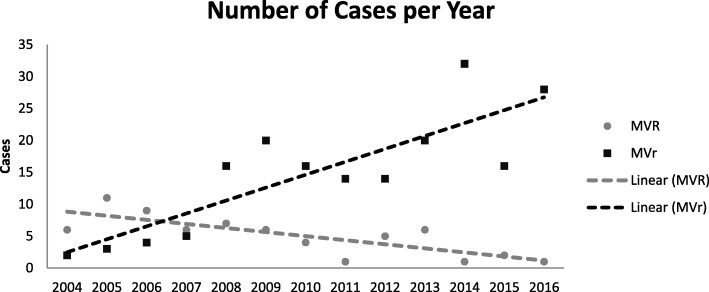
Trends and Number of Cases of Mitral Valve Repair and Replacement. Tendencies and number of cases for degenerative mitral valve repair and replacement between 2004 and 2016. MVr: Mitral Valve Repair MVR: Mitral Valve Replacement

**Fig. 5:**
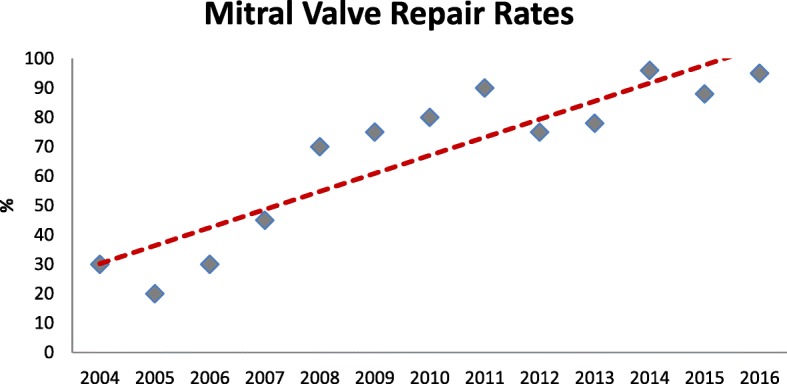
Mitral Valve Repair Rate. The figure shows mitral valve repair rates over the years, tendency line is shown in red

Tendencies and number of cases of MVr and MVR for degenerative MV disease are shown in Fig. 4. Within four years, we inverted the tendency and were able to maintain MVr as preferred technique of MV intervention. The MVr rates at our institution are shown in Fig. 5. Over the years there has been a constant increase in MVr rate, achieving a 96% repair rate in 2016.

## Discussion

MV regurgitation is frequently caused by degenerative MV disease leading to myxomatous changes with chordal elongation with or without rupture [6–8]. R and NR techniques have shown excellent results, with low incidence of progression to severe MR and need for MVR [7–9]. In our series, five patients required MVR after an average of 5.3 years, three belonged to the NR group and two to the R group, one patient from the NR group had an ePTFE chord rupture. Schwartz et al. [10] described similar results with a freedom from reoperation of 89% at ten years. There was no 30-day mortality in our series; Lange et al. [11] showed comparable results with 30-day mortality of 1%. We were able to achieve MVr results with R and NR techniques similar to those reported by referral institutions, despite being a low-volume center.

NR techniques, like chordal replacement, preserve leaflet mobility increasing coaptation surface and avoiding outflow tract obstruction. How to standardize length of the neochordae and the long-term durability of the reapir remain subjects of debate [11, 12]. In our series survival rates of NR techniques for severe MR were 77% (CI 95% 0.38–0.93) at 6.6 years of follow-up and freedom from reoperation was 98.40%. Salvador et al. [13] reported 608 consecutive MVr with NR techniques, with a freedom from reoperation of 92% after 15 years.

R techniques have exhibited excellent results [1, 11], however, these techniques sometimes sacrifice a large amount of valve tissue, resulting in leaflet restriction, and requires a skilled and experienced surgeon. New techniques, like butterfly resection, have been shown to prevent systolic anterior motion, decreasing the need for annular plication [14, 15]. In our series survival rates of R techniques for severe MR were 92.4% (CI 95% 0.69–0.98) at 8.3 years of follow-up, with a freedom from reoperation of 96%. Sakamoto et al. [16] reported the long-term results of this techniques, with a freedom from reoperation of 92,3% at 10 years.

In the matter of functional class, the results are excellent; the majority of patients showed considerable improvement after surgery. In our series, at last follow-up, 156 (82,9%) were in NYHA class I and 181 patients (86%) showed no or grade I MR, with no difference between groups. Lange et al. [11] described similar results, at last follow-up 94% of their patients showed no or grade I MR. The literature supports that the incidence of severe MR, need for reoperation, and death are equally low with R and NR techniques [11, 17–21]. However, the institutions were these investigations were conducted had high-volumes of MVr. It was uncertain if centers with low-volume could reproduce these results.

In our bivariate analysis, we found that patients that underwent minimally invasive repair had a lower risk of developing recurrent moderate MR. This could be explained by the fact that in our practice, minimally invasive MVr is performed by a single surgeon (JPU), who also has the most experience. Further analysis has also shown, that minimally invasive MVr has resulted in earlier referral of patients by cardiologists, leading to patients being healthier, with less comorbidities. Since the NR group had more minimally invasive repairs, this could explain the difference in euroscore II assessments between groups.

Our results show that, despite low volumes in the earlier years of our experience, MVr results achieved can be comparable to those reported by referral centers worldwide, leading to an inversion in the tendency of MVR vs MVr in our Institution and an excellent MVr rates. We attribute this change to the creation of a MVr program, with a dedicated team lead by a MV surgeon (JPU) resulting in better patient selection, standardization of processes and procedures, education of referring physicians, earlier patient referral, and better postoperative care and follow-up.

To improve volume and results of the MVr program, we began to encourage targeted referral and guideline-based assessment of MV pathology. Cardiology, imaging, and critical care teams were optimally equipped and physicians were trained so an earlier referral could be achieved. All MV cases were analyzed by the MVr program before the procedure, and the repair was performed by an experienced surgeon. Cardiac anesthesiologists in charge of the cases were fully prepared to perform echocardiograms in the operating room so the quality of the MVr could be assessed before the patient was weaned of CPB. Junior cardiac surgeons were mentored and technically supported. A valvular heart clinic was created so MV patients could be properly followed and controlled.

With target and earlier referral, we improved patient selection and MVr rates. We were able to operate healthier patients, with less comorbidities, better functional class, younger, and with better LVEF. This was a key factor for achieving and maintaining good results, since patients with previous myocardial infarction, dyslipidemia, dialysis, and hypertension have an increased risk of developing at least moderate recurrent MR, as shown before in different studies [20–23]. The literature has suggested a close relationship between preoperative comorbidities and the odds of developing recurrent MR. Fukuda et al. [24] found a close relationship between type 2 diabetes and the progression of MR. We performed an exploratory logistic binary regression, finding that previous myocardial infarction by itself increases the risk up to 18% and can be modified in the presence of variables such as age, gender, and surgical approach.

Different articles [25, 26] have shown that individual surgeon volume is a determinant of MVr rates, freedom from reoperation, and survival. A total of < 25 MVr per year has been associated with poor results and low MVr rates. When no volume-outcome relationships were available, the United Kingdom proposed a volume threshold of 25 MVr/year for surgeon, so better results could be achieved. In the United States, there is no minimum volume standardized for MVr [26]. At our institution, since the creation of the MVr program, patient volume has grown and MVr rate has improved. We have been able to maintain MVr as preferred technique of MV intervention, and satisfactory results have been obtained. With the creation of a well prepared, well equipped and experienced MVr program, that has a guideline-assessment of MV pathology and is lead by an experienced MV surgeon, adequate MVr results can be accomplished in low-volume centers.

Daneshmand et al. [25] conducted a 20-year study, and concluded that MVr patients have better survival and functional outcomes, especially after 10–15 years, compared to MVR. In keeping with this, Gammie et al. [27] presented the trends of MV surgery in the United States, showing progressive adoption of MVr. In Latin America, however, trends of MV surgery remain unknown, with little data showing trends in MVr vs MVR and different studies have suggested the number of MVr should be increased [27].

This paper has some limitations, it was a retrospective study performed over a period of 15 years. Changes in surgical techniques and postoperative management of the patients might have affected the incidence of recurrent MR.

## Conclusions

In conclusion, short and long-term results with either the R or the NR techniques are equivalent. Recurrence of severe MR and the need for MVR are rare. Significant symptomatic improvement can be achieved in more than 80% of the patients, and the majority will present with no or grade I MR. Risk factors for MR after surgery should be analyzed. The most reliable and durable repair technique for degenerative MV disease is the one that the surgeon feels more comfortable and has the most experience with. This study shows how low-volume centers can achieve results comparable to those reported worldwide as recently suggested by Bakaeen et al. [28]. We attribute the results presented in this paper to the creation of a MVr team, with a dedicated MVr surgeon as the leader.

## Abbreviations

ePTFE: Polytetrafluoroethylene
MR: Mitral regurgitation
MV: Mitral valve
MVr: Mitral valve repair
MVR: Mitral valve replacement
NR: No resection
NYHA: New York Heart Association
R: Resection
